# Learning from Imbalanced Data: Integration of Advanced Resampling Techniques and Machine Learning Models for Enhanced Cancer Diagnosis and Prognosis

**DOI:** 10.3390/cancers16193417

**Published:** 2024-10-08

**Authors:** Fatih Gurcan, Ahmet Soylu

**Affiliations:** 1 Department of Management Information Systems, Faculty of Economics and Administrative Sciences, Karadeniz Technical University, 61080 Trabzon, Turkey; fgurcan@ktu.edu.tr; 2 Department of Computer Science, Faculty of Information Technology and Electrical Engineering, Norwegian University of Science and Technology, 2815 Gjøvik, Norway

**Keywords:** cancer diagnosis and prognosis, class imbalance, machine learning, resampling techniques, random forest, predictive modeling

## Abstract

**Simple Summary:**

This research focuses on improving cancer diagnosis and prognosis by addressing a common problem in data analysis known as class imbalance, where some patient groups are underrepresented. The authors aim to evaluate different resampling methods that can balance the data and enhance the performance of various classification algorithms used to predict cancer outcomes. By testing a wide range of techniques across multiple cancer datasets, this study identifies the best-performing classifier, Random Forest, along with the most effective resampling method, SMOTEENN. These findings provide valuable insights for researchers and healthcare professionals, enabling them to make more accurate predictions and ultimately improve patient care. This research could pave the way for the development of more reliable machine learning applications in the medical field.

**Abstract:**

Background/Objectives: This study aims to evaluate the performance of various classification algorithms and resampling methods across multiple diagnostic and prognostic cancer datasets, addressing the challenges of class imbalance. Methods: A total of five datasets were analyzed, including three diagnostic datasets (Wisconsin Breast Cancer Database, Cancer Prediction Dataset, Lung Cancer Detection Dataset) and two prognostic datasets (Seer Breast Cancer Dataset, Differentiated Thyroid Cancer Recurrence Dataset). Nineteen resampling methods from three categories were employed, and ten classifiers from four distinct categories were utilized for comparison. Results: The results demonstrated that hybrid sampling methods, particularly SMOTEENN, achieved the highest mean performance at 98.19%, followed by IHT (97.20%) and RENN (96.48%). In terms of classifiers, Random Forest showed the best performance with a mean value of 94.69%, with Balanced Random Forest and XGBoost following closely. The baseline method (no resampling) yielded a significantly lower performance of 91.33%, highlighting the effectiveness of resampling techniques in improving model outcomes. Conclusions: This research underscores the importance of resampling methods in enhancing classification performance on imbalanced datasets, providing valuable insights for researchers and healthcare professionals. The findings serve as a foundation for future studies aimed at integrating machine learning techniques in cancer diagnosis and prognosis, with recommendations for further research on hybrid models and clinical applications.

## 1. Introduction

Cancer is considered one of the leading health issues with the highest mortality rates globally. It can arise in various organs and tissues, with each type possessing unique characteristics and treatment approaches. Additionally, cancer cells often exhibit a tendency to spread rapidly, making early diagnosis and effective treatment challenging. A significant contributing factor to cancer’s high mortality rate is that the disease is often diagnosed at advanced stages. Late diagnosis limits treatment options and frequently leads to less successful outcomes. In modern medicine, diagnosing and prognosing cancer typically requires the analysis of large and complex datasets [1,2]. These datasets may include a wide range of information such as patients’ genetic data, clinical test results, imaging data, and biomarkers [3,4]. Effectively diagnosing, managing, treating, and predicting the course of cancer necessitates thorough analysis of complex data from various contexts [1,4,5]. This data complexity mandates the use of robust data analysis and modeling techniques to achieve accurate and reliable results [6,7,8]. Labeled data are data where each instance is associated with a specific target value or class, which is used for training supervised learning algorithms [9,10,11]. Unlabeled data, on the other hand, are data without classification or target value information, typically used for unsupervised learning methods [12,13,14]. The selection of suitable analysis techniques is crucial for both types of data, as it directly impacts the reliability of results and the performance of the model [6,8]. Proper and comprehensive data analysis allows for early-stage diagnosis, the personalization of treatment methods, and more accurate prognoses [1,4,6,14,15].

However, the imbalances commonly encountered in these complex datasets significantly hinder the effectiveness of analysis processes [16,17]. In particular, situations where minority classes (e.g., rare types of cancer) are underrepresented compared to majority classes (e.g., common types of cancer) are frequently observed [11,15,18]. Data imbalance arises when there is a substantial difference in the number of samples between classes, and the underrepresentation of minority classes complicates the models’ ability to learn them accurately [8,19]. This can result in low sensitivity and high false negative rates for minority classes. Models that place more weight on the majority class may not adequately learn the minority classes, leading to an increase in misclassification rates [6,7,11]. Issues stemming from such imbalanced data distributions can adversely affect the performance of traditional classification algorithms, particularly hindering the accurate prediction of minority classes, which are critical in cancer diagnosis and prognosis [20,21].

To overcome these data imbalances, it is essential to develop specific strategies and techniques for data analysis and modeling processes [6,7]. Resampling methods and balancing techniques play a crucial role in this context. These techniques are implemented to ensure better representation of minority classes in the dataset and support classification algorithms in producing fairer and more accurate results [21,22,23]. Resampling methods include various strategies such as reducing the majority class samples (under-sampling), increasing the minority class samples (over-sampling), and smoothing methods that combine these two approaches [7,16,24]. These approaches facilitate dataset balancing, allowing classification algorithms to generate more accurate and reliable results [6,11,23].

The aim of this study is to thoroughly examine how resampling techniques are utilized in cancer diagnosis and prognosis and to compare the performance of these techniques in conjunction with various classifiers. In this context, while evaluating the effectiveness of resampling methods, we compared classification techniques based on various machine learning approaches designed to enhance performance in imbalanced datasets. The findings obtained can contribute to understanding the effects of resampling methods on medical data, improving clinical decision support systems, enhancing the overall quality of healthcare services, and more effectively addressing imbalanced datasets.

## 2. Materials and Methods

This study aims to identify the most effective resampling methods and classifiers for cancer diagnosis and prognosis, addressing the challenge of imbalanced data that can distort model performance. To achieve this, we have structured our methodology in a detailed and systematic manner, which is outlined in the following sequential subsections:

- Description of the datasets: An overview of the datasets used in this study, including their characteristics and relevance to cancer diagnosis and prognosis.
- Adopted data resampling strategies: Overview of techniques used to address data imbalance and their implementation.
- Adopted machine learning classifiers: Summary of algorithms applied, including their purpose and usage.
- Performance evaluation metrics: The criteria and methods used to assess the effectiveness of the classifiers and resampling methods.
- Proposed methodology and experimental setup: An outline of the overall methodological framework and experimental design.

### 2.1. Description of the Datasets

In this study, five datasets focused on diagnosis and prognosis have been used, including three diagnostic and two prognostic datasets. The diagnostic datasets are the Wisconsin Breast Cancer Database [25], which classifies tumors as benign or malignant, the Cancer Prediction Dataset [26], which includes clinical and biological data for various cancer types, and the Lung Cancer Detection Dataset [27], which assesses lung cancer risk using demographic and clinical variables. The prognostic datasets are the Seer Breast Cancer Dataset [28], which predicts long-term survival and cancer progression, and the Differentiated Thyroid Cancer Recurrence Dataset [29], which estimates the likelihood of cancer recurrence in patients with thyroid cancer.

All of these datasets are open-source and publicly available, ensuring transparency and accessibility for research purposes. Researchers can freely access and download these datasets via the Kaggle data repository, using the provided links. These datasets exhibit significant class imbalance problems, with minority classes being underrepresented. This imbalance can negatively affect the performance of machine learning models. Resampling methods, which are used to correct such imbalances, help balance class distribution and improve the accuracy of the models. Each of these datasets will now be explained in detail under individual subsections.

- Wisconsin Breast Cancer Database (Diagnostic): The Wisconsin Breast Cancer Database (WBCD) is a widely recognized dataset in the field of medical research and machine learning, particularly used for breast cancer diagnosis [25]. Each sample is described by 10 real-valued features, which include characteristics such as clump thickness, size and shape uniformity, marginal adhesion, epithelial cell size, and others that are critical for distinguishing between benign and malignant breast masses. These features are derived from digitized images of fine-needle aspirates (FNAs) of breast tissue. The primary objective when using this dataset is to predict the class of the tumor (benign or malignant) based on the given features. The dataset comprises 699 samples, with 458 instances of benign tumors (65.5%) and 241 instances of malignant tumors (34.5%) [6,24,25].
- Cancer Prediction Dataset (Diagnostic): This dataset contains health and lifestyle information for 1500 patients, with 9 variables (Age, Gender, BMI, Smoking, GeneticRisk, PhysicalActivity, AlcoholIntake, CancerHistory, Diagnosis), aimed at predicting cancer diagnosis [26]. It presents a realistic challenge for predictive modeling in the medical field by encompassing a range of variables that influence cancer diagnosis and prognosis. The dataset includes both categorical and continuous features, such as age, BMI, smoking status, and genetic risk factors, which collectively contribute to the complexity of predicting cancer presence. Each variable captures different aspects of patients’ health and lifestyle, adding layers of intricacy to the modeling process [17,23]. Additionally, the dataset includes information on patients’ cancer history and current cancer diagnosis status. In this dataset, 943 patients (62.9%) have not been diagnosed with cancer, while 557 patients (37.1%) have been diagnosed with cancer [26].
- Lung Cancer Detection Dataset (Diagnostic): This dataset contains information related to lung cancer risk factors and symptoms for individuals, with the aim of detecting the presence of lung cancer [27]. It comprises 309 samples with 16 variables that capture a range of demographic, lifestyle, and medical factors linked to lung cancer risk. These Variables Include Gender, Age, Smoking, Yellow_Fingers, Anxiety, Peer_Pressure, Chronic_Disease, Fatigue, Allergy, Wheezing, Alcohol_Consuming, Coughing, Shortness_Of_Breath, Swallowing_Difficulty, Chest_Pain, and Lung_cancer. Each variable provides valuable insights into potential indicators of lung cancer, contributing to the overall analysis. The target variable, Lung_cancer, represents the diagnostic outcome, where 270 individuals (87.4%) are diagnosed with lung cancer, and 39 individuals (12.6%) are not [27]. This imbalance ratio indicates that for every individual without lung cancer, there are about 7 individuals with lung cancer [7,11,20].
- Seer Breast Cancer Dataset (Prognostic): The Seer Breast Cancer Dataset offers comprehensive clinical and demographic information crucial for evaluating breast cancer outcomes [28]. The dataset encompasses 4024 patients, with exclusions made for cases with unknown tumor size, unexamined regional lymph nodes, positive regional lymph nodes, and survival times less than one month. This dataset includes a total of 16 variables, providing a broad spectrum of information: Age, Race, Marital Status, T Stage, N Stage, 6th Stage, Differentiation, Grade, A Stage, Tumor Size, Estrogen Status, Progesterone Status, Regional Node Examined, Regional Node Positive, Survival Months, and Status. The target variable, Status, represents the patient’s survival outcome and is categorized as “Alive” or “Dead”. In this dataset, 84.4% (3398 patients) are classified as “Alive”, while 15.6% (607 patients) are classified as “Dead” [28]. This significant class imbalance highlights a key challenge in prognosis prediction [4,6,7].
- Differentiated Thyroid Cancer Recurrence Dataset (Prognostic): This dataset encompasses important features and information for assessing prognosis in well-differentiated thyroid cancer. The Differentiated Thyroid Cancer Recurrence dataset contains a total of 17 variables and includes 383 patient records [29]. Out of these, 13 are clinicopathologic features: Age, Gender, Smoking, Hx Smoking (history of smoking), Hx Radiotherapy (history of radiotherapy), Thyroid Function, Physical Examination, Adenopathy, Pathology, Focality, Risk, T (tumor stage), N (nodal stage), M (metastasis stage), Stage (overall stage), and Response (treatment response). The target variable, Recurred shows that 275 patients (71.8%) are classified as “No”, meaning they did not experience recurrence, while 108 patients (28.2%) are classified as “Yes”, indicating that their cancer recurred [29]. This substantial imbalance between the two classes presents a significant challenge for predictive modeling [7,20].

### 2.2. Adopted Data Resampling Strategies

In this study, we employ a total of 19 resampling methods, categorized into three types: over-sampling (6 methods), under-sampling (10 methods), and hybrid sampling (2 methods). Additionally, the Baseline scenario, which involves no resampling, is included for comparative purposes [30,31]. For over-sampling, aimed at increasing the number of minority class samples, we use KMSMOTE, SMOTE, ADASYN, ROS, BSMOTE, and SVMSMOTE [7,32,33]. Under sampling methods, which focus on reducing the number of majority class samples to balance the dataset, include IHT, RENN, EditedNN, NCR, TomekLinks, RUS, NearMiss, ClusterC, OSS, and CNN [1,23,30]. Hybrid sampling techniques, which combine both over- and under-sampling methods, are represented by SMOTEENN and SMOTETomek [1,34]. The Baseline scenario, with no resampling, serves as a reference to evaluate the effectiveness of the resampling methods [24]. Each approach is selected to address class imbalance and enhance the performance of machine learning models.

1. Over-Sampling Methods
  - ROS (Random Over-Sampling): This technique balances class distribution by randomly replicating samples from the minority class [18,35].
  - SMOTE (Synthetic Minority Over-Sampling Technique): New examples are generated by interpolating between existing minority class samples and their neighbors [23].
  - ADASYN (Adaptive Synthetic Sampling): This method creates additional synthetic examples by giving more weight to difficult-to-learn minority samples [36].
  - BSMOTE (Borderline-SMOTE): New synthetic examples are generated using minority class samples that are close to the class boundaries [4].
  - KMSMOTE (KMeans SMOTE): This method combines KMeans clustering with SMOTE to replicate minority class samples [37].
  - SVMSMOTE (Support Vector Machine SMOTE): This method generates samples close to the boundaries of the minority class using Support Vector Machines [3,17].
2. Under-Sampling Methods
  - RUS (Random Under-Sampling): Balances the dataset by randomly removing samples from the majority class [7,18].
  - TomekLinks: Removes majority class samples identified as Tomek Links, which are considered to be boundary or noisy examples [1,38].
  - NearMiss: Selects majority class samples that are closest to minority class examples to balance the dataset [1,39].
  - ClusterC (Cluster Centroids): Uses k-means clustering to divide the majority class into clusters and balances the dataset using the cluster centroids [34,40].
  - ENN (Edited Nearest Neighbors): Removes incorrectly classified majority class samples using k-nearest neighbors (k-NN) [23,41].
  - RENN (Repeated Edited Nearest Neighbors): Refines the ENN method by iteratively removing majority class samples to clean the dataset further [41].
  - CNN (Condensed Nearest Neighbor): Reduces the majority class samples by iteratively partitioning the dataset into subsets using the nearest neighbor algorithm [42].
  - IHT (Instance Hardness Threshold): Identifies and removes difficult-to-classify samples from the dataset [10,20].
  - OSS (One-Sided Selection): Uses 1-nearest neighbor (1-NN) to select minority class samples and incorrectly classified majority samples, and then removes boundary majority examples using Tomek Links [4,23].
  - NCR (Neighborhood Cleaning Rule): Employs a three-nearest neighbor rule to remove incorrectly classified majority class samples [1,43].
3. Combination Methods (Over-Sampling and Under-Sampling)
  - SMOTETomek: Combines SMOTE and Tomek Links methods; first generates minority class samples, then cleans noisy and boundary examples [1,38,44].
  - SMOTEENN: Merges SMOTE and ENN methods; generates minority class samples and then removes misclassified examples [1,4,44].

### 2.3. Adopted Machine Learning Classifiers

In this study, we utilize 10 different machine learning classifiers, categorized into four main groups: Balancing Ensemble Classifiers, Linear Classifiers, Standard Ensemble Classifiers, and Deep Learning Classifiers [1,7,45]. This diverse set of classifiers allows for a thorough exploration of machine learning approaches, especially in the context of imbalanced data in cancer diagnosis and prognosis, where achieving accurate predictions is critical for patient outcomes [46,47]. By utilizing a combination of traditional linear models, advanced ensemble techniques, and deep learning classifiers, we aim to assess the effectiveness of each method in addressing the challenges posed by class imbalance. This holistic approach provides valuable insights into the performance and adaptability of different algorithms in predicting cancer progression and aiding early diagnosis.

1. Balancing Ensemble Classifiers:
  - BRF (Balanced Random Forest): Modifies the traditional Random Forest by using balanced datasets during training to handle class imbalance [48,49].
  - EE (Easy Ensemble): Uses multiple bootstrap samples of the minority class and trains independent models, combining their results to create a more balanced classification [16,31].
  - RB (Random Under-Sampling Boost): Applies random under-sampling followed by AdaBoost to provide a balanced classification [24,50].
  - BB (Balanced Bagging): Employs balanced resampling in each bootstrap sample to enhance the performance of the bagging method on imbalanced data [48,51].
2. Linear Classifiers:
  - LR (Logistic Regression): A linear model that predicts class probabilities using a sigmoid function [1,44].
  - SVC (Support Vector Classifier): Finds the optimal hyperplane for classification and supports linear and non-linear separations using different kernels [17,52].
3. Standard Ensemble Classifiers:
  - RF (Random Forest): A popular bagging method that creates multiple decision trees, combining their outputs to improve robustness and reduce overfitting [9,48].
  - XGB (XGBoost): An optimized gradient boosting method that builds trees iteratively to correct previous errors, known for its high performance in large datasets [9,51].
4. Deep Learning Classifiers:
  - MLP (Multi-Layer Perceptron): A feedforward neural network with fully connected layers, effective for solving non-linear classification problems [4,53].
  - DNN (Deep Neural Network): An extension of MLP with multiple hidden layers, allowing for more advanced feature learning and higher capacity in handling large and complex datasets [46,54].

### 2.4. Performance Evaluation Metrics

In this study, we utilize a comprehensive set of five performance evaluation metrics to assess our machine learning models. Accuracy provides a straightforward measure of overall prediction correctness, while the F1 Score balances precision and recall, offering a nuanced evaluation especially important for imbalanced datasets [9,53,55]. ROC-AUC evaluates the model’s ability to distinguish between classes, which is crucial for tasks such as differentiating between benign and malignant tumors [20,56]. The Mean of these metrics, which integrates Accuracy, F1 Score, and ROC-AUC, offers a consolidated view of model performance, reflecting overall effectiveness [50,57,58]. This diverse set of metrics ensures a thorough evaluation of model performance, addressing various aspects crucial for effective cancer diagnosis and prognosis [9,50,59,60]. Class Count refers to the number of instances in each class within the dataset. This is crucial for understanding the distribution and balance of classes [20,33]. The Imbalance Ratio is calculated by dividing the count of the minority class by the count of the majority class, then multiplying by 100 to express it as a percentage. This ratio indicates how prevalent the minority class is relative to the majority class [11,16].

### 2.5. Proposed Methodology and Experimental Setup

This section describes the methodology of this study, which includes six key components: Experimental Setup, Data Loading, Encoding, and Scaling; Application of Resampling Techniques; Application of Classifiers; Performance Evaluation Metrics; and Results Analysis. The sequential stages of this methodology are detailed below under the following subheadings.

- Experimental Setup: The experimental setup for this study was carried out in a stable and efficient computing environment. The system configuration included an Intel i7-12650H processor, Nvidia RTX 3060 GPUs, and 16 GB of RAM, running on Windows 10, 64-bit. Python 3.12 (64-bit) was used as the development language, with Jupyter Notebook version 7.1.3 serving as the primary environment [59]. Each phase of our methodology was supported by specific tools and libraries [49,50]. Python, with its extensive ecosystem, was selected due to its flexibility, scalability, and wide use in machine learning and data science. Its comprehensive library offerings enabled efficient management of every stage of the machine learning workflow, from data preparation to model evaluation and deployment [8,49,61,62,63]. The source codes used in this study are publicly available on Figshare [64].
- Data Loading, Encoding, and Scaling: The datasets used in this study were loaded from CSV files and prepared for analysis. Invalid values were replaced with NaN and filled using the most frequently repeated values for each respective column. The dependent and target variables were identified and extracted based on the specific objectives of this study [50]. Non-numeric target variables were converted into numerical format using Label Encoding, while features were scaled using Standard Scaling [3,16]. Data splitting was performed using 5-Fold Cross-Validation, where the dataset was divided into five equal parts [50,65]. Each fold served as a test set while the remaining four folds were used for training, ensuring that each instance of the dataset was used for both training and testing. This method preserves the class distribution in each fold and provides a comprehensive evaluation of the model’s performance [49,50,65].
- Application of Resampling Methods: To reduce class imbalance and enhance model performance, a total of 19 different resampling techniques were applied across three categories: over-sampling, under-sampling, and hybrid methods [1,4,20,38,44]. Also, a Baseline model without resampling was used for performance comparison. The dataset was processed using these methods to create balanced training sets, which were then used to evaluate the performance of various classifiers [18,20,34]. After resampling, the balanced training sets were used to train various classifiers [49,50,65].
- Application of Classifiers: Various classifiers, encompassing a total of ten models, were applied to the resampled datasets, and these were systematically categorized into four key groups—Balancing Ensemble Classifiers [48], Linear Classifiers [54], Standard Ensemble Classifiers [9], and Deep Learning Classifiers [17,58]—each contributing to a comprehensive performance evaluation. Each classifier was tested on the resampled datasets to evaluate its performance [66]. Additionally, Early Stopping was used for deep learning models to prevent overfitting [49,50,61,65].
- Performance Evaluation and Result Analysis: The performance of the classifiers was assessed using metrics such as accuracy, F1 score, ROC-AUC, and the mean of these metrics [11,60]. All experiments were conducted using the Stratified K-Fold (5-Fold) Cross-Validation method [1,9,67]. Performance results for each resampling method and classifier combination were collected and analyzed. The results were summarized using metrics such as accuracy, F1 score, ROC AUC, Mean, imbalance ratio, and class counts [6,7,52].

## 3. Results and Discussion

### 3.1. Results for Wisconsin Breast Cancer Database (Diagnostic)

In this study, the impact of class imbalance on classification performance and the effectiveness of various resampling methods were assessed using the Wisconsin Breast Cancer (Diagnostic) dataset. Resampling methods significantly improved these results, as detailed in Table 1. Initially, the dataset had an imbalance ratio of 52.62%, limiting model performance. The IHT method yielded the highest performance with a Mean value of 99.73%, 99.59% Accuracy, 99.59% F1 Score, and 99.99% ROC AUC. SMOTEENN followed closely, with the same Mean (99.73%) and slightly higher scores of 99.61% for both Accuracy and F1 and 99.96% ROC AUC. RENN and EditedNN also performed well, with Mean values of 99.41% and 99.20%, respectively. The Baseline approach lagged behind with a 97.20% Mean, reflecting the impact of imbalance. As shown in Table 1, resampling methods, especially IHT and SMOTEENN, significantly enhanced performance. However, under-sampling methods like OSS and CNN led to reduced accuracy, highlighting the need for carefully chosen resampling techniques to mitigate class imbalance.

Table 2 presents the performance of ten different classification algorithms applied to the Wisconsin Breast Cancer Database (Diagnostic) using the resampling methods mentioned above. Multi-Layer Perceptron (MLP) achieved the best performance with a mean value of 97.83%, excelling with 97.41% Accuracy, 97.39% F1 Score, and 98.68% ROC AUC, effectively distinguishing both classes. Random Forest ranked second with a mean of 97.80%. Logistic Regression placed third with a mean of 97.79%, particularly standing out with a 98.94% ROC AUC, highlighting its ability to distinguish positive classes. Balanced Bagging, at the bottom of the list, showed relatively lower performance with a mean of 96.60%, but still achieved strong classification with a 98.08% ROC AUC. This analysis demonstrates that MLP, Random Forest, and Logistic Regression provided the best performance, with strong class separation shown by their ROC AUC values.

### 3.2. Results for Cancer Prediction Dataset (Diagnostic)

The classification performance of various resampling methods on the Cancer Prediction Dataset (Diagnostic) is compared in Table 3. Initially, the dataset exhibited a notable class imbalance with an imbalance ratio of 59.07% (0: 943, 1: 557). In the Baseline results, where no resampling methods were applied, the accuracy was 89.59%, the F1 score was 89.54%, and the ROC AUC value was 93.97%. These results indicate that the class imbalance negatively impacted the model’s performance. The RENN method achieved the highest performance with a mean value of 98.67%. Similarly, the hybrid method SMOTEENN also demonstrated strong performance, with 97.90% accuracy, 97.90% F1 score, and 99.65% ROC AUC. Hybrid methods, such as SMOTEENN and SMOTETomek, effectively managed class imbalance by combining both over-sampling and under-sampling techniques. The IHT method yielded a mean value of 95.07%. However, under-sampling methods like NearMiss, ClusterC, OSS, and CNN showed lower mean values. Overall, the results highlight that resampling methods can significantly improve classification performance on imbalanced datasets. Methods such as RENN and SMOTEENN provided the best performance, underscoring the importance of resampling techniques in handling imbalanced data.

Table 4 presents a performance evaluation in which a number of classification algorithms are ranked based on their mean values. The Easy Ensemble model achieved the highest performance, with a mean score of 94.35%. This model provided balanced and effective classification, reaching 93.27% Accuracy, 93.28% F1 Score, and 96.48% ROC AUC. Following this, Balanced Random Forest exhibited strong performance with a mean score of 93.93%, while Random Forest ranked third with a mean score of 93.82%, also delivering robust results. The lowest-performing model was Logistic Regression, which ranked last with a mean score of 88.04%. This model particularly struggled in terms of Accuracy (85.85%) and F1 Score (85.79%). Overall, this evaluation highlights that the Easy Ensemble and Balanced Random Forest models provided the best results, while the other models displayed lower performances in comparison.

### 3.3. Results for Lung Cancer Detection Dataset (Diagnostic)

The experiments conducted on the Lung Cancer Detection Dataset (Diagnostic) highlight the impact of class imbalance on classification performance. The imbalanced distribution within the dataset was addressed through various resampling methods, and the effects of these methods on model performance are presented in Table 5. Among hybrid sampling methods, SMOTEENN stood out with the highest mean value of 99.64%. Similarly, IHT, an under-sampling method, performed well with a mean value of 98.20%. Other methods like RENN and KMSMOTE also delivered notable results, achieving mean values of 97.01% and 96.14%, respectively. However, the Baseline method, which involved classification without any resampling, showed lower performance with a mean value of 89.13%. This result underscores the limitation of model accuracy and overall performance when class imbalance is not addressed. Notably, some under-sampling methods, such as CNN and NearMiss, demonstrated lower performance, achieving mean values of 71.50% and 70.63%, respectively. In conclusion, methods such as SMOTEENN, IHT, and RENN emerged as the most effective approaches for addressing class imbalance and improving classification accuracy.

Table 6 presents the performance of various classification algorithms ranked by their Mean values. The Random Forest model demonstrated the best performance with a Mean value of 93.49%. This model achieved 92.61% Accuracy, 92.44% F1 Score, and 95.43% ROC AUC, delivering strong classification results. Balanced Random Forest ranked second with a high performance, achieving a Mean value of 93.10%. The Support Vector Classifier (SVC) ranked third with a Mean value of 92.77%, demonstrating strong class distinction with a ROC AUC of 95.03%. Among the lower-performing models, Easy Ensemble achieved a Mean value of 91.02%, while Balanced Bagging and RUSBoost occupied the last positions with Mean values of 89.47% and 89.29%, respectively. These results highlight that the Random Forest and Balanced Random Forest models achieved the best performance, while other models performed slightly lower in comparison.

### 3.4. Results for Seer Breast Cancer Dataset (Prognostic)

The impact of various resampling methods on classification performance is examined using the Seer Breast Cancer Dataset (Prognostic), with results presented in Table 7. The dataset revealed a remarkable class imbalance with an imbalance ratio of 18.08% (0:3408, 1:616), and the effectiveness of various resampling methods in addressing this imbalance was investigated. In the baseline scenario, where no resampling is applied, a Mean value of 84.61% is achieved, indicating lower performance. This result clearly demonstrates the effectiveness of resampling methods in addressing class imbalance. The SMOTEENN method, which performs hybrid sampling, stands out with a Mean value of 94.49%. Among under-sampling methods, IHT exhibits the second-best performance with a Mean value of 94.40%. KMSMOTE is also noteworthy, with a Mean value of 93.50%, demonstrating effective handling of class imbalance. By contrast, some under-sampling methods like CNN and RUS exhibit lower performance. CNN, with a Mean value of 72.24%, ranks at the bottom (see Table 7). The results emphasize that the effective use of resampling methods can substantially improve model performance. Specifically, methods like SMOTEENN, IHT, and KMSMOTE have shown high performance and successful outcomes in classification tasks.

Table 8 presents the performance of various classification algorithms. Random Forest model achieved the best performance, topping the list with a Mean value of 91.14%. Balanced Random Forest ranked second with a Mean value of 90.40%, particularly excelling in ROC AUC performance. XGBoost followed closely in third place, showing similar success with a Mean value of 90.34%. The model with the weakest performance was RUSBoost, which ranked last with a Mean value of 84.56%. Overall, Random Forest and Balanced Random Forest were the top-performing models, while the other models achieved lower results.

### 3.5. Results for Differentiated Thyroid Cancer Recurrence Dataset (Prognostic)

The experiments conducted on the Differentiated Thyroid Cancer Recurrence Dataset (Prognostic) comprehensively demonstrate the impact of various resampling methods on classification performance. In this dataset, class imbalance played a significant role, and the results presented in Table 9 demonstrate how various methods managed this imbalance. The dataset exhibited an imbalance ratio of 39.27% in the baseline scenario, reflecting a notable disparity between classes. For the baseline case, where no resampling method was applied, a Mean value of 94.69% was obtained, indicating that the model’s performance was constrained by the imbalanced data. The best-performing method was SMOTEENN, with a Mean value of 98.60%. IHT followed closely, ranking second with a Mean value of 98.59%. Among other under-sampling methods, RENN stood out with a Mean value of 97.20%. KMSMOTE performed well with a Mean value of 97.18%, while ROS and SMOTETomek also showed strong performances, with Mean values of 97.15% and 96.63%, respectively. By contrast, under-sampling methods such as ClusterC and RUS performed less effectively, with Mean values of 92.06% and 92.03%, demonstrating limited impact on classification tasks. The CNN method recorded the lowest performance with a Mean value of 87.65%.

Table 10 presents the performance of various classification algorithms ranked by their Mean values. The best-performing model is Random Forest, topping the list with a Mean value of 97.21%. XGBoost ranks second with a Mean of 97.06%, notably excelling in ROC AUC with a score of 99.12%. Balanced Random Forest also achieved a Mean of 97.06%, placing third with balanced results across metrics. Lower-performing models include RUSBoost, Support Vector Classifier (SVC), and Multi-Layer Perceptron (MLP), which scored Mean values of 95.29%, 94.25%, and 93.99%, respectively, below the average. Deep Neural Network (DNN) and Logistic Regression showed even lower performance, with Logistic Regression ranking last with a Mean of 91.82%. Overall, Random Forest, XGBoost, and Balanced Random Forest demonstrated the highest performance, while Logistic Regression performed the worst.

### 3.6. Overall Performance Evaluation of the Classifiers and Resampling Methods

**Figure 1:**
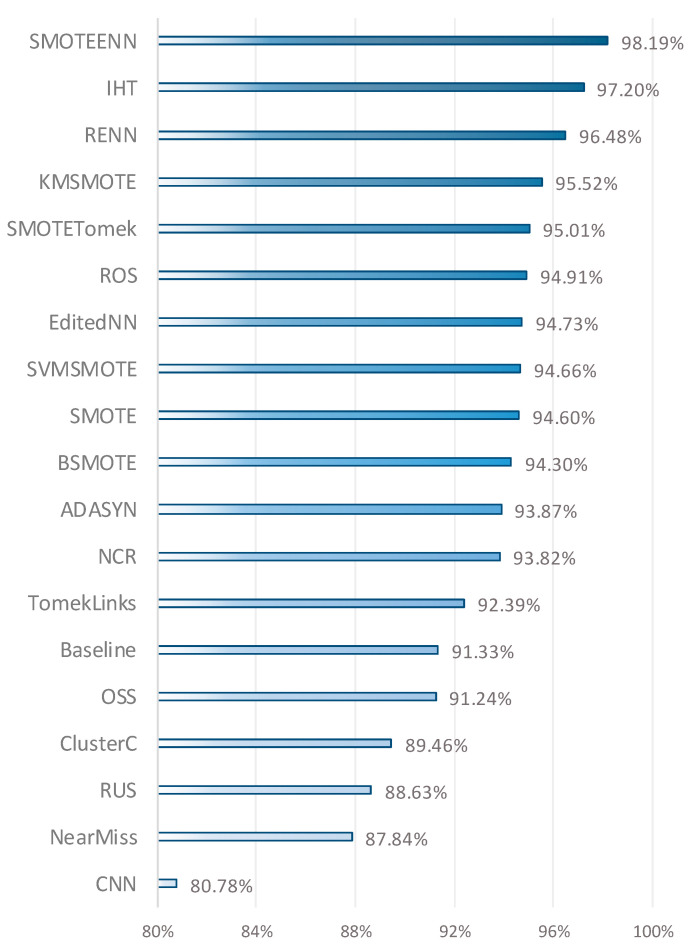
Performance measurements of resampling methods across all datasets.

At this stage of our analysis, we conducted a comprehensive evaluation of the average performance of resampling methods across five datasets: three diagnostic and two prognostics. The Mean value was calculated for each resampling method across all datasets. This approach provides a holistic view of model performance, allowing us to compare the effectiveness of various resampling techniques. Averaging these metrics helps evaluate each method’s performance under different data conditions, enhancing the generalizability of the results. The performance overview in Figure 1 offers a unified perspective on how resampling methods manage class imbalance across all datasets. As shown, SMOTEENN, a hybrid sampling method, achieved the highest performance with a Mean value of 98.19%. IHT ranked second with a Mean value of 97.20%, as an under-sampling method that focuses on identifying and prioritizing more challenging samples. RENN, another under-sampling technique, ranked third with a Mean value of 96.48%. KMSMOTE (an over-sampling method) and SMOTETomek (a hybrid sampling method) followed, with Mean values of 95.52% and 95.01%, respectively. At the lower end of the ranking, RUS, NearMiss, and CNN—all under-sampling methods—recorded Mean values of 88.63%, 87.84%, and 80.78%, showing comparatively lower performances. Overall, the Table highlights the effectiveness of various resampling methods, with SMOTEENN, IHT, and RENN emerging as the top performers. The calculation of Mean values facilitated a comprehensive assessment of classification success across different conditions, offering valuable insight into the most effective methods for managing class imbalance.

The results of the ANOVA and Kruskal–Wallis tests indicate that the performance differences in resampling methods are statistically significant. The ANOVA test produced an F-statistic of 4.3223 and a *p*-value of 0.0181, suggesting that there is a significant difference in performance among at least some of the resampling methods. Similarly, the Kruskal–Wallis test yielded an H-statistic of 10.0332 and a *p*-value of 0.0066. These results emphasize that the performance differences are not due to random variation but rather stem from the effects of different resampling methods on specific datasets and classification tasks.

**Figure 2:**
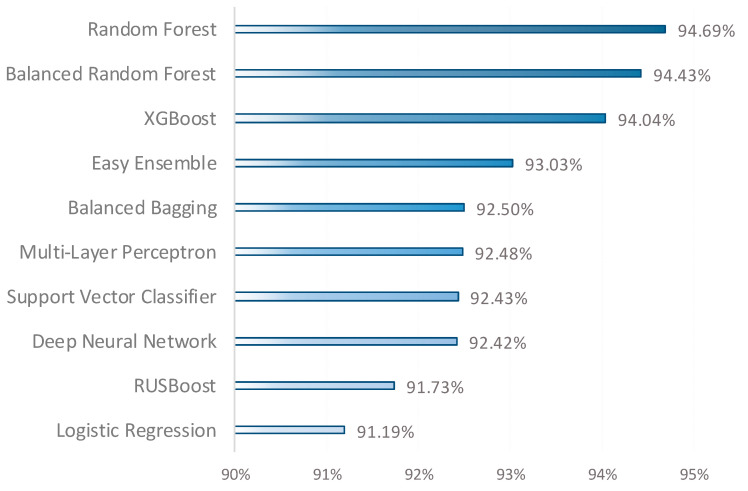
Performance measurements of the classifiers across all datasets.

Figure 2 provides a summary view of each classifier’s performance across the five datasets. As shown, the Random Forest classifier achieved the highest performance, with a Mean value of 94.69%, indicating its robustness and adaptability across different dataset characteristics. Following closely, the Balanced Random Forest, which is designed to handle imbalanced datasets by re-sampling the data during training, recorded a Mean value of 94.43%. XGBoost, another powerful ensemble method, ranked third with a Mean value of 94.04%. At the lower end of the rankings, RUSBoost achieved a Mean value of 91.73%, still showing respectable performance. Lastly, Logistic Regression, a traditional but often reliable classifier, recorded a Mean of 91.19%. Overall, this figure emphasizes the strength of ensemble methods like Random Forest, Balanced Random Forest, and XGBoost, as they consistently ranked highest in terms of average performance.

The results obtained from the ANOVA and Kruskal–Wallis tests clearly indicate that the performance differences among the classifiers are statistically significant. The ANOVA test yielded an F-statistic of 26.6848 and a *p*-value of 0.0000, suggesting that at least one classifier performs significantly differently from the others. Additionally, the Kruskal–Wallis test provided an H-statistic of 19.3617 and a *p*-value of 0.0001, reinforcing the finding of significant differences based on ranking. These findings emphasize that each classifier’s performance varies and that these differences are not due to random chance but rather represent a meaningful effect. Therefore, analyzing performance differences is a crucial step in selecting the most effective classifier.

The results of this study demonstrate that the implemented resampling methods offer an effective strategy for addressing the imbalance between classes, significantly enhancing the performance of the classification models. Resampling methods have facilitated the balancing of representation ratios among classes, ensuring that the minority class is adequately represented. Consequently, this has allowed the models to learn the characteristics of the minority class during the training process, enabling more accurate identification of samples belonging to this class. Moreover, these techniques have reduced the risk of overfitting, leading to the attainment of more generalizable and robust results. By eliminating the excessive representation of the majority class, these methods have prevented the model from focusing solely on a specific class during training, thus allowing for an equitable learning of the characteristics of both classes.

### 3.7. Limitations and Validity

This study presents several limitations that must be considered when interpreting the results. First, the performance of classifiers depends heavily on the quality and diversity of the datasets used. While the selected cancer datasets are reputable, differences in data collection, sample sizes, and demographics may affect generalizability. The datasets primarily feature binary outcome variables and a limited number of predictors, representing only a subset of broader data modeling challenges. Expanding to more diverse datasets with multi-class outcomes and additional features is necessary for broader applicability. Addressing this will be crucial for ensuring more comprehensive and adaptable machine learning solutions in healthcare.

The study focuses on specific resampling techniques and a limited range of classifiers (Balancing Ensemble, Linear, Standard Ensemble, and Deep Learning), which could restrict the findings. Future research should explore a wider array of algorithms, including newer deep learning and hybrid models, to provide a more comprehensive analysis. Additionally, the lack of attention to model interpretability and transparency is a limitation, as understanding model predictions is crucial in clinical settings. Future work should prioritize both model diversity and interpretability to ensure clinical trust and adoption.

To strengthen validation, future work should involve more diverse datasets reflecting different cancer types, stages, and patient demographics. Cross-validation and external validation cohorts mirroring real-world settings would enhance generalizability. Furthermore, incorporating interpretability tools like SHAP or LIME can increase the practical relevance of models in healthcare, ensuring they meet clinical standards. These efforts are key to making machine learning models reliable and applicable in real-world clinical environments.

## 4. Conclusions

In this study, the performance of various classification algorithms and resampling methods was comprehensively evaluated across three diagnostic and two prognostic datasets. The results obtained demonstrate the effectiveness of both machine learning classifiers and resampling techniques in managing the issue of class imbalance. A total of 19 different resampling methods from three distinct categories were employed in this study. SMOTEENN emerged as the most successful method with a mean value of 98.19%, followed by IHT at 97.20%. RENN ranked third with a mean value of 96.48%. By contrast, the baseline (no resampling) method achieved only 91.33%, underscoring the importance of resampling methods in enhancing model performance on imbalanced datasets. The findings of this study illustrate the effectiveness of implementing resampling techniques in addressing the challenges of imbalance and improving classification success.

A total of 10 algorithms from four different categories were utilized as classifiers in this study. According to the obtained findings, Random Forest achieved the highest mean value of 94.69%, demonstrating the effectiveness of the bagging method. Balanced Random Forest followed closely with a mean value of 94.43%, showcasing its capability to handle imbalanced data. XGBoost ranked third with a mean value of 94.04%, recognized for its optimization and high performance as a gradient boosting method. These results emphasize the role of bagging and boosting techniques in enhancing model performance on classification problems with imbalanced datasets. This study provides significant contributions by comprehensively evaluating the performance of various classification algorithms and resampling methods across five different cancer datasets.

The findings highlight the crucial role of resampling methods in improving model performance on imbalanced datasets, offering a foundation for future research. Future studies should assess different classification algorithms and resampling techniques across various datasets, including both diagnostic and prognostic data, to tackle class imbalance comprehensively. Developing hybrid or optimized versions of ensemble and deep learning models could lead to higher accuracy and faster learning. Testing these methods in clinical applications is key to proving their effectiveness in real-world healthcare settings. Additionally, focusing on model interpretability and reliability is essential for ensuring healthcare professionals can safely utilize and trust model outcomes. These recommendations provide a roadmap for enhancing machine learning’s role in healthcare and its integration into clinical practice.

## Figures and Tables

**Table 1:** Performance measurements of resampling methods on the Wisconsin Breast Cancer dataset.

Resampling Method	Sampling Type	Imbalance Ratio	Class Counts	Accuracy	F1 Score	ROC AUC	Mean
IHT	Under-Sampling	64.61%	0: 373, 1: 241	99.59%	99.59%	99.99%	99.73%
SMOTEENN	Hybrid Sampling	98.17%	0: 436, 1: 428	99.61%	99.61%	99.96%	99.73%
RENN	Under-Sampling	55.40%	0: 435, 1: 241	99.14%	99.14%	99.93%	99.41%
EditedNN	Under-Sampling	55.15%	0: 437, 1: 241	98.86%	98.86%	99.88%	99.20%
NCR	Under-Sampling	55.15%	0: 437, 1: 241	98.73%	98.73%	99.87%	99.11%
TomekLinks	Under-Sampling	53.56%	0: 450, 1: 241	97.44%	97.44%	99.47%	98.12%
SMOTETomek	Hybrid Sampling	100.00%	0: 457, 1: 457	97.26%	97.26%	99.26%	97.93%
RUS	Under-Sampling	100.00%	0: 241, 1: 241	97.28%	97.28%	99.20%	97.92%
KMSMOTE	Over-Sampling	99.35%	1: 461, 0: 458	97.13%	97.13%	99.37%	97.87%
SMOTE	Over-Sampling	100.00%	0: 458, 1: 458	97.14%	97.14%	99.32%	97.87%
ADASYN	Over-Sampling	99.34%	0: 458, 1: 455	97.27%	97.27%	98.92%	97.82%
ROS	Over-Sampling	100.00%	0: 458, 1: 458	97.00%	97.00%	99.27%	97.75%
BSMOTE	Over-Sampling	100.00%	0: 458, 1: 458	97.16%	97.16%	98.94%	97.75%
SVMSMOTE	Over-Sampling	100.00%	0: 458, 1: 458	97.15%	97.15%	98.87%	97.72%
Baseline	No Resampling	52.62%	0: 458, 1: 241	96.25%	96.26%	99.10%	97.20%
NearMiss	Under-Sampling	100.00%	0: 241, 1: 241	96.19%	96.18%	98.64%	97.00%
ClusterC	Under-Sampling	100.00%	0: 241, 1: 241	94.81%	94.80%	98.41%	96.01%
OSS	Under-Sampling	19.09%	1: 241, 0: 46	93.34%	93.46%	95.56%	94.12%
CNN	Under-Sampling	12.45%	1: 241, 0: 30	88.96%	89.34%	86.86%	88.39%

**Table 2:** Performance measurements of the classifiers on the Wisconsin Breast Cancer dataset.

Classifier	Accuracy	F1 Score	ROC AUC	Mean
Multi-Layer Perceptron	97.41%	97.39%	98.68%	97.83%
Random Forest	97.49%	97.45%	98.44%	97.80%
Logistic Regression	97.24%	97.20%	98.94%	97.79%
Deep Neural Network	97.19%	97.16%	98.58%	97.65%
Balanced Random Forest	97.06%	97.13%	98.73%	97.64%
XGBoost	96.98%	96.94%	98.24%	97.39%
Support Vector Classifier	97.20%	97.15%	97.73%	97.36%
RUSBoost	96.24%	96.33%	98.53%	97.03%
Easy Ensemble	95.99%	96.16%	98.69%	96.95%
Balanced Bagging	95.78%	95.94%	98.08%	96.60%

**Table 3:** Performance measurements of resampling methods on the Cancer Prediction Dataset.

Resampling Method	Sampling Type	Imbalance Ratio	Class Counts	Accuracy	F1 Score	ROC AUC	Mean
RENN	Under-Sampling	86.57%	0: 551, 1: 477	98.15%	98.15%	99.72%	98.67%
SMOTEENN	Hybrid Sampling	88.07%	1: 746, 0: 657	97.90%	97.90%	99.65%	98.48%
IHT	Under-Sampling	97.55%	0: 571, 1: 557	94.15%	94.14%	96.91%	95.07%
SMOTETomek	Hybrid Sampling	100.00%	1: 905, 0: 905	92.80%	92.79%	96.81%	94.13%
EditedNN	Under-Sampling	83.51%	0: 667, 1: 557	92.13%	92.11%	96.06%	93.43%
NCR	Under-Sampling	76.30%	0: 730, 1: 557	91.92%	91.90%	95.95%	93.25%
ROS	Over-Sampling	100.00%	1: 943, 0: 943	91.61%	91.61%	95.78%	93.00%
KMSMOTE	Over-Sampling	100.00%	1: 943, 0: 943	91.43%	91.42%	95.84%	92.90%
SMOTE	Over-Sampling	100.00%	1: 943, 0: 943	91.27%	91.27%	95.51%	92.68%
TomekLinks	Under-Sampling	62.65%	0: 889, 1: 557	90.64%	90.61%	94.73%	91.99%
OSS	Under-Sampling	63.01%	0: 884, 1: 557	90.60%	90.57%	94.53%	91.90%
SVMSMOTE	Over-Sampling	100.00%	1: 943, 0: 943	90.11%	90.10%	94.93%	91.71%
Baseline	No Resampling	59.07%	0: 943, 1: 557	89.59%	89.54%	93.97%	91.03%
BSMOTE	Over-Sampling	100.00%	1: 943, 0: 943	89.26%	89.26%	94.55%	91.02%
ADASYN	Over-Sampling	92.00%	1: 1025, 0: 943	88.72%	88.71%	94.59%	90.67%
RUS	Under-Sampling	100.00%	0: 557, 1: 557	88.17%	88.16%	93.61%	89.98%
NearMiss	Under-Sampling	100.00%	0: 557, 1: 557	86.65%	86.64%	91.34%	88.21%
ClusterC	Under-Sampling	100.00%	0: 557, 1: 557	86.11%	86.10%	92.10%	88.10%
CNN	Under-Sampling	50.45%	1: 557, 0: 281	82.19%	82.04%	88.16%	84.13%

**Table 4:** Performance measurements of the classifiers on the Cancer Prediction Dataset.

Classifier	Accuracy	F1 Score	ROC AUC	Mean
Easy Ensemble	93.27%	93.28%	96.48%	94.35%
Balanced Random Forest	92.82%	92.82%	96.16%	93.93%
Random Forest	92.65%	92.61%	96.19%	93.82%
XGBoost	92.22%	92.20%	95.97%	93.46%
RUSBoost	90.94%	90.96%	95.58%	92.49%
Multi-Layer Perceptron	90.03%	90.01%	94.48%	91.51%
Balanced Bagging	89.99%	89.99%	94.28%	91.42%
Deep Neural Network	89.80%	89.77%	93.80%	91.12%
Support Vector Classifier	89.47%	89.43%	94.42%	91.11%
Logistic Regression	85.85%	85.79%	92.49%	88.04%

**Table 5:** Performance measurements of resampling methods on the Lung Cancer Detection Dataset.

Resampling Method	Sampling Type	Imbalance Ratio	Class Counts	Accuracy	F1 Score	ROC AUC	Mean
SMOTEENN	Hybrid Sampling	88.93%	0: 253, 1: 225	99.50%	99.50%	99.92%	99.64%
IHT	Under-Sampling	53.42%	1: 73, 0: 39	97.45%	97.44%	99.71%	98.20%
RENN	Under-Sampling	17.11%	1: 228, 0: 39	95.92%	95.98%	99.14%	97.01%
KMSMOTE	Over-Sampling	100.00%	1: 270, 0: 270	95.15%	95.14%	98.14%	96.14%
EditedNN	Under-Sampling	16.67%	1: 234, 0: 39	95.05%	95.16%	98.17%	96.13%
ROS	Over-Sampling	100.00%	1: 270, 0: 270	95.19%	95.17%	97.79%	96.05%
SMOTE	Over-Sampling	100.00%	1: 270, 0: 270	94.85%	94.84%	98.06%	95.92%
SMOTETomek	Hybrid Sampling	100.00%	1: 270, 0: 270	94.85%	94.84%	98.06%	95.92%
SVMSMOTE	Over-Sampling	100.00%	1: 270, 0: 270	94.76%	94.75%	98.03%	95.85%
ADASYN	Over-Sampling	98.89%	1: 270, 0: 267	94.62%	94.61%	97.98%	95.73%
BSMOTE	Over-Sampling	100.00%	1: 270, 0: 270	94.20%	94.19%	98.02%	95.47%
NCR	Under-Sampling	15.98%	1: 244, 0: 39	93.08%	93.28%	97.33%	94.57%
TomekLinks	Under-Sampling	14.72%	1: 265, 0: 39	89.83%	90.04%	92.81%	90.90%
OSS	Under-Sampling	15.73%	1: 248, 0: 39	89.44%	89.95%	93.12%	90.84%
Baseline	No Resampling	14.44%	1: 270, 0: 39	87.85%	88.25%	91.29%	89.13%
ClusterC	Under-Sampling	100.00%	0: 39, 1: 39	86.48%	86.30%	94.44%	89.07%
RUS	Under-Sampling	100.00%	0: 39, 1: 39	83.08%	82.82%	88.49%	84.80%
CNN	Under-Sampling	81.25%	1: 48, 0: 39	70.27%	69.65%	74.58%	71.50%
NearMiss	Under-Sampling	100.00%	0: 39, 1: 39	69.32%	68.96%	73.61%	70.63%

**Table 6:** Performance measurements of the classifiers on the Lung Cancer Detection Dataset.

Classifier	Accuracy	F1 Score	ROC AUC	Mean
Random Forest	92.61%	92.44%	95.43%	93.49%
Balanced Random Forest	91.94%	92.01%	95.35%	93.10%
Support Vector Classifier	91.75%	91.53%	95.03%	92.77%
Logistic Regression	91.45%	91.36%	95.08%	92.63%
Deep Neural Network	91.08%	90.99%	93.89%	91.99%
XGBoost	90.92%	90.76%	94.13%	91.94%
Multi-Layer Perceptron	91.07%	90.94%	93.72%	91.91%
Easy Ensemble	89.20%	89.57%	94.30%	91.02%
Balanced Bagging	87.69%	88.03%	92.69%	89.47%
RUSBoost	88.01%	88.10%	91.77%	89.29%

**Table 7:** Performance measurements of resampling methods on the Seer Breast Cancer Dataset.

Resampling Method	Sampling Type	Imbalance Ratio	Class Counts	Accuracy	F1 Score	ROC AUC	Mean
SMOTEENN	Hybrid Sampling	77.51%	1: 3068, 0: 2378	92.87%	92.88%	97.71%	94.49%
IHT	Under-Sampling	75.77%	0: 813, 1: 616	93.04%	93.01%	97.15%	94.40%
KMSMOTE	Over-Sampling	99.91%	1: 3411, 0: 3408	91.99%	91.99%	96.52%	93.50%
SVMSMOTE	Over-Sampling	100.00%	0: 3408, 1: 3408	89.74%	89.74%	95.48%	91.65%
BSMOTE	Over-Sampling	100.00%	0: 3408, 1: 3408	89.11%	89.11%	94.75%	90.99%
ROS	Over-Sampling	100.00%	0: 3408, 1: 3408	88.82%	88.81%	94.18%	90.60%
SMOTETomek	Hybrid Sampling	100.00%	0: 3380, 1: 3380	88.38%	88.38%	94.56%	90.44%
SMOTE	Over-Sampling	100.00%	0: 3408, 1: 3408	88.11%	88.11%	94.42%	90.21%
RENN	Under-Sampling	27.43%	0: 2246, 1: 616	89.42%	89.24%	91.74%	90.13%
ADASYN	Over-Sampling	97.83%	0: 3408, 1: 3334	87.15%	87.14%	93.37%	89.22%
NearMiss	Under-Sampling	100.00%	0: 616, 1: 616	87.33%	87.29%	92.73%	89.12%
EditedNN	Under-Sampling	23.48%	0: 2624, 1: 616	88.75%	88.56%	88.93%	88.75%
NCR	Under-Sampling	23.07%	0: 2670, 1: 616	86.10%	86.18%	87.51%	86.60%
TomekLinks	Under-Sampling	18.91%	0: 3257, 1: 616	86.86%	86.59%	84.71%	86.05%
OSS	Under-Sampling	18.92%	0: 3255, 1: 616	85.44%	85.36%	84.51%	85.11%
Baseline	No Resampling	18.08%	0: 3408, 1: 616	85.59%	85.23%	83.00%	84.61%
ClusterC	Under-Sampling	100.00%	0: 616, 1: 616	79.33%	79.30%	87.49%	82.04%
RUS	Under-Sampling	100.00%	0: 616, 1: 616	75.88%	75.83%	83.62%	78.44%
CNN	Under-Sampling	76.33%	0: 807, 1: 616	71.33%	70.94%	74.45%	72.24%

**Table 8:** Performance measurements of the classifiers on the Seer Breast Cancer Dataset.

Classifier	Accuracy	F1 Score	ROC AUC	Mean
Random Forest	90.49%	90.21%	92.73%	91.14%
Balanced Random Forest	89.18%	89.28%	92.73%	90.40%
XGBoost	89.65%	89.44%	91.91%	90.34%
Balanced Bagging	87.40%	87.49%	90.86%	88.58%
Deep Neural Network	86.81%	86.72%	88.60%	87.37%
Multi-Layer Perceptron	86.22%	86.02%	89.22%	87.15%
Support Vector Classifier	85.60%	85.11%	89.24%	86.65%
Easy Ensemble	84.01%	84.33%	90.62%	86.32%
Logistic Regression	84.30%	83.99%	88.76%	85.68%
RUSBoost	82.26%	82.50%	88.93%	84.56%

**Table 9:** Performance measurements of resampling methods on the Differentiated Thyroid Cancer Recurrence Dataset.

Resampling Method	Sampling Type	Imbalance Ratio	Class Counts	Accuracy	F1 Score	ROC AUC	Mean
SMOTEENN	Hybrid Sampling	97.93%	1: 242, 0: 237	98.12%	98.12%	99.55%	98.60%
IHT	Under-Sampling	76.06%	0: 142, 1: 108	98.04%	98.02%	99.71%	98.59%
RENN	Under-Sampling	46.15%	0: 234, 1: 108	96.57%	96.54%	98.49%	97.20%
KMSMOTE	Over-Sampling	99.28%	1: 277, 0: 275	96.32%	96.32%	98.89%	97.18%
ROS	Over-Sampling	100.00%	0: 275, 1: 275	96.22%	96.22%	99.01%	97.15%
SMOTETomek	Hybrid Sampling	100.00%	0: 272, 1: 272	95.53%	95.53%	98.82%	96.63%
SVMSMOTE	Over-Sampling	100.00%	0: 275, 1: 275	95.24%	95.23%	98.63%	96.37%
SMOTE	Over-Sampling	100.00%	0: 275, 1: 275	95.13%	95.12%	98.78%	96.34%
BSMOTE	Over-Sampling	100.00%	0: 275, 1: 275	95.07%	95.07%	98.66%	96.27%
EditedNN	Under-Sampling	44.63%	0: 242, 1: 108	95.14%	95.10%	98.18%	96.14%
ADASYN	Over-Sampling	96.00%	0: 275, 1: 264	94.71%	94.71%	98.32%	95.91%
NCR	Under-Sampling	47.37%	0: 228, 1: 108	94.51%	94.46%	97.73%	95.57%
TomekLinks	Under-Sampling	40.15%	0: 269, 1: 108	93.61%	93.58%	97.44%	94.88%
Baseline	No Resampling	39.27%	0: 275, 1: 108	93.71%	93.64%	96.74%	94.69%
NearMiss	Under-Sampling	100.00%	0: 108, 1: 108	92.65%	92.62%	97.45%	94.24%
OSS	Under-Sampling	42.52%	0: 254, 1: 108	92.87%	92.80%	96.96%	94.21%
ClusterC	Under-Sampling	100.00%	0: 108, 1: 108	90.20%	90.16%	95.84%	92.06%
RUS	Under-Sampling	100.00%	0: 108, 1: 108	89.83%	89.78%	96.49%	92.03%
CNN	Under-Sampling	44.44%	1: 108, 0: 48	85.34%	85.42%	92.20%	87.65%

**Table 10:** Performance measurements of the classifiers on the Differentiated Thyroid Cancer Recurrence Dataset.

Classifier	Accuracy	F1 Score	ROC AUC	Mean
Random Forest	96.38%	96.38%	98.89%	97.21%
XGBoost	96.03%	96.02%	99.12%	97.06%
Balanced Random Forest	96.12%	96.13%	98.93%	97.06%
Easy Ensemble	95.28%	95.30%	98.88%	96.49%
Balanced Bagging	95.53%	95.53%	98.18%	96.41%
RUSBoost	93.98%	93.99%	97.90%	95.29%
Support Vector Classifier	92.69%	92.66%	97.39%	94.25%
Multi-Layer Perceptron	92.51%	92.47%	97.00%	93.99%
Deep Neural Network	92.86%	92.77%	96.21%	93.95%
Logistic Regression	90.09%	90.04%	95.34%	91.82%

## Data Availability

The datasets used in this research are publicly available, and access links are provided as references in the dataset section of the article.
